# Tricarbon­yl(η^6^-4′,7-dimethoxy­iso­flavone)chromium(0)

**DOI:** 10.1107/S1600536809040537

**Published:** 2009-10-10

**Authors:** Johannes H. van Tonder, Barend C. B. Bezuidenhoudt, J. Marthinus Janse van Rensburg

**Affiliations:** a Department of Chemistry, University of the Free State, PO Box 339, Bloemfontein 9300, South Africa; b Department of Pharmacology, University of Pretoria, PO Box 2034, Pretoria 0001, South Africa

## Abstract

The metal atom of the Cr(CO)_3_ unit of the title compound, [Cr(C_17_H_14_O_4_)(CO)_3_], is coordinated to the methoxy­phenyl ring of the isoflavone ligand; the Cr(CO)_3_ unit exhibits a three-legged piano-stool conformation. The aromatic ring of the methoxy­phenyl group is twisted by 42.49 (9)° with respect to the γ-pyrone ring. In the fused-ring, the dihedral angle between the phenyl­ene and γ-pyrone rings is 3.08 (13)°.

## Related literature

For tricarbon­yl(arene)chromium complexes in regioselective reactions, see: Dominique *et al.* (1999 ▶). For their photochromic properties, see: Hannesschlager *et al.* (1999 ▶). For Cr(CO)_3_ complexation to the aromatic ring of flavanone, see: Dominique *et al.* (1999 ▶). For Cr(CO)_3_ complexation to (1,3-dimeth­oxy­benzene), see: Zeller *et al.* (2004 ▶). For comparison bond distances, see: Allen (2002 ▶). For the synthesis of 4′,7-dimethoxy­isoflavone, see: Thakkar & Cushman (1995 ▶).
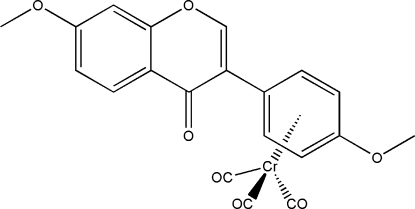


## Experimental

### 

#### Crystal data

[Cr(C_17_H_14_O_4_)(CO)_3_]
*M* *_r_* = 418.31
Monoclinic, 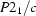

*a* = 12.3454 (7) Å
*b* = 17.9984 (8) Å
*c* = 7.9988 (4) Å
β = 103.733 (2)°
*V* = 1726.50 (15) Å^3^
*Z* = 4
Mo *K*α radiation
μ = 0.71 mm^−1^
*T* = 173 K
0.43 × 0.23 × 0.10 mm

#### Data collection

Bruker APEXII CCD diffractometer
Absorption correction: multi-scan (*SADABS*; Bruker, 2004 ▶) *T*_min_ = 0.751, *T*_max_ = 0.933
9320 measured reflections
4145 independent reflections
3393 reflections with *I* > 2σ(*I*)
*R*_int_ = 0.027

#### Refinement

*R*[*F* ^2^ > 2σ(*F* ^2^)] = 0.044
*wR*(*F* ^2^) = 0.117
*S* = 1.08
4145 reflections
255 parameters
H-atom parameters constrained
Δρ_max_ = 0.86 e Å^−3^
Δρ_min_ = −0.41 e Å^−3^

### 

Data collection: *APEX2* (Bruker, 2005 ▶); cell refinement: *SAINT-Plus* (Bruker, 2004 ▶); data reduction: *SAINT-Plus*; program(s) used to solve structure: *SIR97* (Altomare *et al.*, 1999 ▶); program(s) used to refine structure: *SHELXL97* (Sheldrick, 2008 ▶); molecular graphics: *DIAMOND* (Brandenburg & Putz, 2005 ▶); software used to prepare material for publication: *WinGX* (Farrugia, 1999 ▶).

## Supplementary Material

Crystal structure: contains datablocks global, I. DOI: 10.1107/S1600536809040537/ng2656sup1.cif

Structure factors: contains datablocks I. DOI: 10.1107/S1600536809040537/ng2656Isup2.hkl

Additional supplementary materials:  crystallographic information; 3D view; checkCIF report

## Figures and Tables

**Fig. 1.**
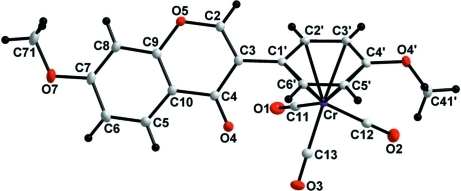
A view of (I) showing the atom-numbering scheme with displacement ellipsoids at the 30% probability level.

**Fig. 2.**
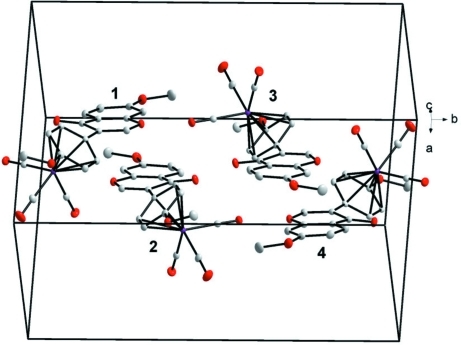
Indication of molecular packing in the unit-cell. Symmetry operators 1) x; y; z. 2) x; 0.5 - y; -1/2 + z. 3) 1 - x; 1/2 + y; 1.5 - z. 4) 1 - x; 1 - y; 1 - z.

## supplementary crystallographic information

### Comment

Tricarbonyl(arene)chromium complexes have recieved much attention due to their use as intermediates in regioselective reactions (Dominique *et al.*, 1999), as well as for their photochromic properties (Hannesschlager *et al.*, 1999).

The title compound, (I), [Cr(CO)_3_(C_17_H_14_O_4_)], where (C_17_H_14_O_4_) = 4',7-dimethoxyisoflavone, crystallized in the monoclinic space group *P*2_1_/*c*, with *Z* = 4 (Fig.1). For the title compound the molecular structure displays the Cr(CO)_3_ moiety complexation to the phenyl ring, exhibiting the known three-legged piano-stool conformation. This conformation is expected for a tricarbonyl-metal with an η^6^-coordinated arene. The Cr—C(arene) distances range from 2.188 (2) to 2.262 (2) Å. The longest Cr—C(arene) bond is Cr—C4', that in turn is bonded to the O4'—C41' methoxy group. This bond elongation is probably due to the methoxy group that weakens the π-interaction ability of C4' towards the chromium metal centre. The Cr-arene(centroid) distance is 1.7205 (4) Å. The Cr—*C*(carbonyl) bond distances range from 1.827 (3) to 1.855 (3) Å and the carbonyl distances of C11—O1, C12—O2 and C13—O3 are 1.158 (3), 1.154 (3) and 1.150 (3) Å respectively. These distances are within the normal range, see Allen (2002). The phenyl ring is essentialy planar (r.m.s of fitted atoms C1'-C6' = 0.0119 Å). Slight molecular disorder is displayed by a twist in the isoflavone backbone, that forms a dihedral angle of 42.49 (9)° between the phenyl and γ-pyrone ring and a dihedral angle of 41.1 (1)° between the phenyl and the benzopyrone ring system. A dihedral angle of 3.08 (13)° is also present between the benzene and the γ-pyrone ring, with a r.m.s of fitted atoms C2—C10 and O5 of 0.0387 Å. The O4'—C41' methoxy group on the phenyl ring bends towards the Cr(CO)_3_ moiety, forming the C5'-C4'-O4'-C41' tortion angle of 15.9 (4)°. The O7—C71 methoxy group on the benzene ring is also slightly displaced from the benzene ring plane, shown by the C8—C7—O7—C71 tortion angle of 175.0 (3)°. Other molecular geometrical parameters is in good agreement with literature values, see Allen (2002). Selected geometrical parameters is presented in Table 1.

As illustrated in Fig.2 the molecular packing is such that a benzene ring of one molecule is above the γ-pyrone ring of a neighbouring molecule, separated by a plane to plane distance of 3.369 Å and a centroid to centroid distance of 4.281 Å.

### Experimental

4',7-Dimethoxyisoflavone was prepared as previously described by Thakkar & Cushman (1995). A solution of 4',7-Dimethoxyisoflavone (1.28 g, 4.5 mmol) and Cr(CO)_6_ (1.00 g, 4.6 mmol: 1 eq.) in Bu_2_O:THF (9:1; 10 ml per 100 mg Cr(CO)_6_ was degassed with argon, using standard Schlenk techniques, and refluxed (48 h) under an oxygen free atmosphere. The reaction mixture was cooled to room temperature and the solvent evaporated *in vacuo*. Purification through flash column-chromatography yielded tricarbonyl(B-η^6^-4',7-dimethoxyisoflavone)-chromium(0) (0.48 g; 25.0%) as a yellow solid. Recrystallization from diethyl ether yielded yellow cuboidal crystals.

*R_f_* 0.18 (Hexane: Acetone; 8:2); Mp 127.0 °C; Note: A, B and C-ring labelling refers to the benzene, phenyl and γ-pyrone rings respectively. ^1^H NMR (600 MHz, CDCl_3_) δ p.p.m. 8.15 (1*H*, d, J = 9.04 Hz, H-5), 8.09 (1*H*, s, H-2), 7.01 (1*H*, dd, J = 1.88, 9.04 Hz, H-6), 6.86 (1*H*, d, J = 1.88 Hz, H-8), 5.85 (2*H*, d, J = 6.78 Hz, H-2' and H-6'), 5.21 (2*H*, d, J = 6.78 Hz, H-3' and H-5'), 3.92 (3*H*, s, –O*CH_3_*), 3.75 (3*H*, s, –O*CH_3_*); ^13^C NMR (151 MHz, CDCl_3_) δ p.p.m. 55.88 (–O*CH_3_*), 56.06 (–O*CH_3_*), 77.37 (C-3' and C-5'), 94.71, 97.63 (C-2' and C-6'), 100.45 (C-8), 115.32 (C-6), 117.67, 121.16, 127.71 (C-5), 143.39 (C(i)-OCH_3_ B-ring), 154.78 (C-2), 158.07, 164.60, 175.26 (C-4), 232.89 (Cr—CO); MS (MS Scheme 3) m/*z* 362 (*M*^+^-2CO, 0.5%), 343 (2.1), 282 (100.0), 267 (20.8), 252 (3.0), 239 (10.9), 224 (3.7), 211 (3.8), 196 (3.5), 183 (1.2), 168 (2.9), 150 (12.9), 141 (6.1), 131 (69.5), 122 (10.7), 107 (7.9), 103 (2.4).

### Refinement

The H atoms were positioned geometrically and refined using a riding model with fixed C—H distances of 0.93 Å (CH) [*U*_iso_(H) = 1.2*U*_eq_] and 0.96 Å (CH_3_) [*U*_iso_(H) = 1.5*U*_eq_] respectively. Initial positions of methyl H-atoms were obtained from fourier difference and refined as a fixed rotor.

The highest density peak is 0.86 located 0.96 Å from O1 and the deepest hole is -0.41 located at 0.54 Å from Cr.

### Crystal data

**Table:**

[Cr(C_17_H_14_O_4_)(CO)_3_]	*F*(000) = 856
*M**_r_* = 418.31	*D*_x_ = 1.609 Mg m^−^^3^
Monoclinic, *P*2_1_/*c*	Mo *K*α radiation, λ = 0.71073 Å
Hall symbol: -P 2ybc	Cell parameters from 3261 reflections
*a* = 12.3454 (7) Å	θ = 2.8–28.3°
*b* = 17.9984 (8) Å	µ = 0.71 mm^−^^1^
*c* = 7.9988 (4) Å	*T* = 173 K
β = 103.733 (2)°	Block, yellow
*V* = 1726.50 (15) Å^3^	0.43 × 0.23 × 0.1 mm
*Z* = 4	

### Data collection

**Table:**

Bruker APEXII CCD diffractometer	4145 independent reflections
Radiation source: fine-focus sealed tube	3393 reflections with *I* > 2σ(*I*)
graphite	*R*_int_ = 0.027
φ and ω scans	θ_max_ = 28°, θ_min_ = 1.7°
Absorption correction: multi-scan (*SADABS*; Bruker, 2004)	*h* = −16→9
*T*_min_ = 0.751, *T*_max_ = 0.933	*k* = −23→21
9320 measured reflections	*l* = −10→9

### Refinement

**Table:**

Refinement on *F*^2^	0 restraints
Least-squares matrix: full	H-atom parameters constrained
*R*[*F*^2^ > 2σ(*F*^2^)] = 0.044	*w* = 1/[σ^2^(*F*_o_^2^) + (0.0453*P*)^2^ + 2.2801*P*] where *P* = (*F*_o_^2^ + 2*F*_c_^2^)/3
*wR*(*F*^2^) = 0.117	(Δ/σ)_max_ < 0.001
*S* = 1.08	Δρ_max_ = 0.86 e Å^−^^3^
4145 reflections	Δρ_min_ = −0.41 e Å^−^^3^
255 parameters	

### Special details

**Table:**

Experimental. The intensity data was collected on a Bruker Apex II CCD diffractometer using an exposure time of 10 s/frame. The 509 frames were collected with a frame width of 0.5° covering up to θ = 28° with 99.4% completeness accomplished.
Geometry. All e.s.d.'s (except the e.s.d. in the dihedral angle between two l.s. planes) are estimated using the full covariance matrix. The cell e.s.d.'s are taken into account individually in the estimation of e.s.d.'s in distances, angles and torsion angles; correlations between e.s.d.'s in cell parameters are only used when they are defined by crystal symmetry. An approximate (isotropic) treatment of cell e.s.d.'s is used for estimating e.s.d.'s involving l.s. planes.

### Fractional atomic coordinates and isotropic or equivalent isotropic displacement parameters (Å^2^)

**Table:**

	*x*	*y*	*z*	*U*_iso_*/*U*_eq_	
Cr	0.67866 (3)	0.08506 (2)	0.83271 (5)	0.01520 (12)	
C71	−0.0137 (3)	0.34474 (18)	0.1743 (5)	0.0415 (8)	
H71A	−0.0177	0.3606	0.29	0.062*	
H71B	−0.0845	0.3559	0.0926	0.062*	
H71C	0.0469	0.3713	0.1402	0.062*	
C1'	0.5514 (2)	0.16695 (13)	0.8698 (3)	0.0170 (5)	
C2	0.4317 (2)	0.26583 (14)	0.7185 (3)	0.0210 (5)	
H2	0.4859	0.2986	0.7838	0.025*	
C2'	0.6553 (2)	0.20306 (14)	0.8800 (3)	0.0198 (5)	
H2'	0.6591	0.2432	0.8046	0.024*	
C3'	0.7524 (2)	0.18039 (14)	0.9997 (3)	0.0212 (5)	
H3'	0.8208	0.2058	1.0066	0.025*	
C3	0.4495 (2)	0.19286 (14)	0.7448 (3)	0.0185 (5)	
C4	0.3661 (2)	0.13983 (14)	0.6510 (3)	0.0192 (5)	
C4'	0.7479 (2)	0.11970 (14)	1.1093 (3)	0.0201 (5)	
C5	0.1963 (2)	0.13098 (15)	0.4082 (4)	0.0248 (6)	
H5	0.2022	0.0784	0.4126	0.03*	
C5'	0.6471 (2)	0.08121 (14)	1.0971 (3)	0.0182 (5)	
H5'	0.6442	0.0395	1.1688	0.022*	
C6	0.1094 (2)	0.16347 (16)	0.2927 (4)	0.0272 (6)	
H6	0.0567	0.1336	0.2156	0.033*	
C6'	0.5506 (2)	0.10521 (14)	0.9775 (3)	0.0185 (5)	
H6'	0.4828	0.0789	0.9692	0.022*	
C7	0.0986 (2)	0.24107 (16)	0.2887 (4)	0.0246 (6)	
C8	0.1774 (2)	0.28543 (15)	0.3938 (4)	0.0241 (6)	
H8	0.1713	0.338	0.3893	0.029*	
C9	0.2667 (2)	0.25058 (14)	0.5070 (3)	0.0196 (5)	
C10	0.2769 (2)	0.17415 (14)	0.5204 (3)	0.0196 (5)	
C11	0.6772 (2)	0.11149 (14)	0.6116 (4)	0.0224 (5)	
C12	0.8108 (2)	0.03640 (15)	0.8470 (4)	0.0252 (6)	
C13	0.6030 (2)	−0.00087 (14)	0.7453 (3)	0.0213 (5)	
C41'	0.8552 (3)	0.03005 (17)	1.2974 (4)	0.0326 (7)	
H41A	0.8072	0.0277	1.3788	0.049*	
H41B	0.9328	0.0212	1.3587	0.049*	
H41C	0.8319	−0.0079	1.2082	0.049*	
O1	0.6770 (2)	0.12958 (12)	0.4726 (3)	0.0362 (5)	
O2	0.89489 (18)	0.00706 (14)	0.8540 (3)	0.0461 (6)	
O3	0.55781 (18)	−0.05454 (11)	0.6900 (3)	0.0318 (5)	
O4'	0.84603 (16)	0.10203 (11)	1.2190 (2)	0.0260 (4)	
O4	0.36907 (17)	0.07292 (10)	0.6784 (3)	0.0271 (4)	
O5	0.34364 (15)	0.29706 (10)	0.6077 (2)	0.0227 (4)	
O7	0.00685 (17)	0.26705 (12)	0.1746 (3)	0.0347 (5)	

### Atomic displacement parameters (Å^2^)

**Table:**

	*U*^11^	*U*^22^	*U*^33^	*U*^12^	*U*^13^	*U*^23^
Cr	0.0162 (2)	0.01354 (19)	0.0156 (2)	−0.00044 (15)	0.00332 (14)	0.00014 (15)
C71	0.0309 (16)	0.0381 (18)	0.050 (2)	0.0135 (14)	−0.0004 (15)	0.0107 (15)
C1'	0.0199 (12)	0.0159 (11)	0.0154 (11)	0.0018 (9)	0.0048 (9)	−0.0032 (9)
C2	0.0205 (12)	0.0201 (12)	0.0211 (12)	0.0020 (10)	0.0024 (10)	0.0009 (10)
C2'	0.0242 (13)	0.0139 (11)	0.0208 (12)	−0.0011 (9)	0.0047 (10)	−0.0009 (9)
C3'	0.0204 (12)	0.0188 (12)	0.0228 (13)	−0.0029 (10)	0.0020 (10)	−0.0026 (10)
C3	0.0185 (12)	0.0199 (12)	0.0177 (12)	0.0012 (9)	0.0058 (10)	0.0018 (10)
C4	0.0187 (12)	0.0188 (12)	0.0203 (12)	0.0014 (9)	0.0054 (10)	0.0015 (10)
C4'	0.0218 (12)	0.0192 (12)	0.0173 (12)	0.0002 (10)	0.0007 (10)	−0.0037 (10)
C5	0.0249 (13)	0.0200 (13)	0.0277 (14)	−0.0016 (10)	0.0028 (11)	0.0021 (11)
C5'	0.0272 (13)	0.0156 (11)	0.0124 (11)	0.0003 (10)	0.0060 (9)	0.0014 (9)
C6	0.0227 (13)	0.0291 (14)	0.0268 (14)	−0.0044 (11)	0.0002 (11)	0.0029 (12)
C6'	0.0191 (12)	0.0193 (12)	0.0179 (12)	−0.0008 (9)	0.0059 (10)	−0.0012 (9)
C7	0.0173 (12)	0.0293 (14)	0.0269 (14)	0.0042 (10)	0.0049 (10)	0.0086 (11)
C8	0.0241 (13)	0.0209 (13)	0.0268 (14)	0.0044 (10)	0.0049 (11)	0.0062 (11)
C9	0.0187 (12)	0.0215 (12)	0.0190 (12)	0.0012 (10)	0.0051 (10)	0.0006 (10)
C10	0.0187 (12)	0.0198 (12)	0.0209 (12)	0.0004 (9)	0.0056 (10)	0.0027 (10)
C11	0.0237 (13)	0.0178 (12)	0.0247 (14)	−0.0014 (10)	0.0038 (10)	−0.0025 (10)
C12	0.0245 (13)	0.0227 (13)	0.0260 (14)	0.0002 (11)	0.0013 (11)	−0.0061 (11)
C13	0.0242 (13)	0.0210 (13)	0.0199 (12)	−0.0013 (10)	0.0073 (10)	0.0001 (10)
C41'	0.0289 (15)	0.0312 (15)	0.0324 (16)	0.0028 (12)	−0.0033 (12)	0.0096 (13)
O1	0.0576 (15)	0.0321 (11)	0.0212 (10)	−0.0032 (10)	0.0140 (10)	0.0037 (9)
O2	0.0268 (12)	0.0449 (14)	0.0615 (16)	0.0122 (10)	0.0002 (11)	−0.0138 (12)
O3	0.0364 (12)	0.0229 (10)	0.0373 (12)	−0.0090 (9)	0.0110 (9)	−0.0063 (9)
O4'	0.0216 (9)	0.0258 (10)	0.0255 (10)	−0.0009 (7)	−0.0041 (8)	0.0029 (8)
O4	0.0275 (10)	0.0177 (9)	0.0312 (11)	−0.0030 (7)	−0.0030 (8)	0.0048 (8)
O5	0.0232 (9)	0.0172 (9)	0.0253 (10)	0.0024 (7)	0.0009 (8)	0.0008 (7)
O7	0.0233 (10)	0.0340 (12)	0.0410 (13)	0.0039 (8)	−0.0038 (9)	0.0103 (10)

### Geometric parameters (Å, °)

**Table:**

Cr—C11	1.827 (3)	C4—C10	1.463 (3)
Cr—C12	1.831 (3)	C4'—O4'	1.354 (3)
Cr—C13	1.855 (3)	C4'—C5'	1.407 (4)
Cr—C2'	2.188 (2)	C5—C6	1.369 (4)
Cr—C6'	2.200 (2)	C5—C10	1.405 (4)
Cr—C1'	2.225 (2)	C5—H5	0.95
Cr—C3'	2.231 (3)	C5'—C6'	1.407 (3)
Cr—C5'	2.241 (2)	C5'—H5'	0.95
Cr—C4'	2.262 (2)	C6—C7	1.403 (4)
C71—O7	1.421 (4)	C6—H6	0.95
C71—H71A	0.98	C6'—H6'	0.95
C71—H71B	0.98	C7—O7	1.358 (3)
C71—H71C	0.98	C7—C8	1.379 (4)
C1'—C6'	1.407 (3)	C8—C9	1.398 (4)
C1'—C2'	1.423 (3)	C8—H8	0.95
C1'—C3	1.484 (3)	C9—O5	1.374 (3)
C2—C3	1.340 (4)	C9—C10	1.383 (4)
C2—O5	1.351 (3)	C11—O1	1.158 (3)
C2—H2	0.95	C12—O2	1.154 (3)
C2'—C3'	1.406 (4)	C13—O3	1.150 (3)
C2'—H2'	0.95	C41'—O4'	1.432 (3)
C3'—C4'	1.410 (4)	C41'—H41A	0.98
C3'—H3'	0.95	C41'—H41B	0.98
C3—C4	1.472 (3)	C41'—H41C	0.98
C4—O4	1.223 (3)		
			
C11—Cr—C12	89.38 (12)	C4'—C3'—Cr	72.89 (14)
C11—Cr—C13	87.92 (12)	C2'—C3'—H3'	120.2
C12—Cr—C13	89.19 (12)	C4'—C3'—H3'	120.2
C11—Cr—C2'	86.74 (11)	Cr—C3'—H3'	129.5
C12—Cr—C2'	127.26 (11)	C2—C3—C4	119.1 (2)
C13—Cr—C2'	143.03 (11)	C2—C3—C1'	119.7 (2)
C11—Cr—C6'	128.55 (11)	C4—C3—C1'	121.2 (2)
C12—Cr—C6'	141.88 (11)	O4—C4—C10	121.9 (2)
C13—Cr—C6'	88.62 (10)	O4—C4—C3	124.0 (2)
C2'—Cr—C6'	66.94 (10)	C10—C4—C3	114.0 (2)
C11—Cr—C1'	96.39 (11)	O4'—C4'—C5'	124.7 (2)
C12—Cr—C1'	162.83 (11)	O4'—C4'—C3'	115.1 (2)
C13—Cr—C1'	107.12 (10)	C5'—C4'—C3'	120.2 (2)
C2'—Cr—C1'	37.61 (9)	O4'—C4'—Cr	129.89 (18)
C6'—Cr—C1'	37.09 (9)	C5'—C4'—Cr	70.96 (14)
C11—Cr—C3'	106.73 (11)	C3'—C4'—Cr	70.53 (15)
C12—Cr—C3'	95.71 (11)	C6—C5—C10	121.1 (3)
C13—Cr—C3'	164.55 (11)	C6—C5—H5	119.4
C2'—Cr—C3'	37.07 (9)	C10—C5—H5	119.4
C6'—Cr—C3'	78.48 (10)	C6'—C5'—C4'	119.2 (2)
C1'—Cr—C3'	67.15 (9)	C6'—C5'—Cr	69.97 (14)
C11—Cr—C5'	163.24 (11)	C4'—C5'—Cr	72.62 (14)
C12—Cr—C5'	106.15 (11)	C6'—C5'—H5'	120.4
C13—Cr—C5'	98.36 (10)	C4'—C5'—H5'	120.4
C2'—Cr—C5'	78.88 (9)	Cr—C5'—H5'	129.3
C6'—Cr—C5'	36.93 (9)	C5—C6—C7	119.7 (3)
C1'—Cr—C5'	66.93 (9)	C5—C6—H6	120.2
C3'—Cr—C5'	66.21 (9)	C7—C6—H6	120.2
C11—Cr—C4'	142.14 (11)	C5'—C6'—C1'	122.1 (2)
C12—Cr—C4'	86.93 (11)	C5'—C6'—Cr	73.10 (14)
C13—Cr—C4'	129.64 (11)	C1'—C6'—Cr	72.42 (14)
C2'—Cr—C4'	66.30 (9)	C5'—C6'—H6'	119
C6'—Cr—C4'	65.90 (9)	C1'—C6'—H6'	119
C1'—Cr—C4'	78.58 (9)	Cr—C6'—H6'	127.7
C3'—Cr—C4'	36.57 (9)	O7—C7—C8	124.4 (3)
C5'—Cr—C4'	36.43 (9)	O7—C7—C6	114.7 (3)
O7—C71—H71A	109.5	C8—C7—C6	120.9 (2)
O7—C71—H71B	109.5	C7—C8—C9	117.9 (2)
H71A—C71—H71B	109.5	C7—C8—H8	121
O7—C71—H71C	109.5	C9—C8—H8	121
H71A—C71—H71C	109.5	O5—C9—C10	121.5 (2)
H71B—C71—H71C	109.5	O5—C9—C8	115.8 (2)
C6'—C1'—C2'	117.5 (2)	C10—C9—C8	122.7 (2)
C6'—C1'—C3	122.2 (2)	C9—C10—C5	117.6 (2)
C2'—C1'—C3	120.2 (2)	C9—C10—C4	121.0 (2)
C6'—C1'—Cr	70.49 (14)	C5—C10—C4	121.4 (2)
C2'—C1'—Cr	69.80 (14)	O1—C11—Cr	178.7 (2)
C3—C1'—Cr	129.11 (17)	O2—C12—Cr	178.4 (3)
C3—C2—O5	126.0 (2)	O3—C13—Cr	178.8 (2)
C3—C2—H2	117	O4'—C41'—H41A	109.5
O5—C2—H2	117	O4'—C41'—H41B	109.5
C3'—C2'—C1'	121.2 (2)	H41A—C41'—H41B	109.5
C3'—C2'—Cr	73.13 (15)	O4'—C41'—H41C	109.5
C1'—C2'—Cr	72.59 (14)	H41A—C41'—H41C	109.5
C3'—C2'—H2'	119.4	H41B—C41'—H41C	109.5
C1'—C2'—H2'	119.4	C4'—O4'—C41'	117.5 (2)
Cr—C2'—H2'	126.9	C2—O5—C9	117.9 (2)
C2'—C3'—C4'	119.7 (2)	C7—O7—C71	117.4 (2)
C2'—C3'—Cr	69.80 (14)		
			
C11—Cr—C1'—C6'	153.08 (16)	C5'—Cr—C4'—O4'	119.8 (3)
C12—Cr—C1'—C6'	−97.9 (4)	C11—Cr—C4'—C5'	152.16 (18)
C13—Cr—C1'—C6'	63.30 (16)	C12—Cr—C4'—C5'	−122.82 (17)
C2'—Cr—C1'—C6'	−130.8 (2)	C13—Cr—C4'—C5'	−36.4 (2)
C3'—Cr—C1'—C6'	−101.42 (16)	C2'—Cr—C4'—C5'	103.89 (16)
C5'—Cr—C1'—C6'	−28.66 (14)	C6'—Cr—C4'—C5'	29.69 (14)
C4'—Cr—C1'—C6'	−64.94 (15)	C1'—Cr—C4'—C5'	66.45 (15)
C11—Cr—C1'—C2'	−76.17 (16)	C3'—Cr—C4'—C5'	133.3 (2)
C12—Cr—C1'—C2'	32.8 (4)	C11—Cr—C4'—C3'	18.8 (2)
C13—Cr—C1'—C2'	−165.95 (15)	C12—Cr—C4'—C3'	103.85 (17)
C6'—Cr—C1'—C2'	130.8 (2)	C13—Cr—C4'—C3'	−169.74 (16)
C3'—Cr—C1'—C2'	29.33 (15)	C2'—Cr—C4'—C3'	−29.44 (15)
C5'—Cr—C1'—C2'	102.09 (16)	C6'—Cr—C4'—C3'	−103.64 (17)
C4'—Cr—C1'—C2'	65.82 (15)	C1'—Cr—C4'—C3'	−66.88 (16)
C11—Cr—C1'—C3	36.9 (2)	C5'—Cr—C4'—C3'	−133.3 (2)
C12—Cr—C1'—C3	145.9 (4)	O4'—C4'—C5'—C6'	−180.0 (2)
C13—Cr—C1'—C3	−52.9 (2)	C3'—C4'—C5'—C6'	−1.6 (4)
C2'—Cr—C1'—C3	113.1 (3)	Cr—C4'—C5'—C6'	−54.1 (2)
C6'—Cr—C1'—C3	−116.1 (3)	O4'—C4'—C5'—Cr	−125.9 (3)
C3'—Cr—C1'—C3	142.4 (2)	C3'—C4'—C5'—Cr	52.5 (2)
C5'—Cr—C1'—C3	−144.8 (2)	C11—Cr—C5'—C6'	34.8 (4)
C4'—Cr—C1'—C3	178.9 (2)	C12—Cr—C5'—C6'	−167.93 (16)
C6'—C1'—C2'—C3'	−3.3 (4)	C13—Cr—C5'—C6'	−76.34 (16)
C3—C1'—C2'—C3'	178.8 (2)	C2'—Cr—C5'—C6'	66.23 (15)
Cr—C1'—C2'—C3'	−56.9 (2)	C1'—Cr—C5'—C6'	28.78 (14)
C6'—C1'—C2'—Cr	53.6 (2)	C3'—Cr—C5'—C6'	102.91 (16)
C3—C1'—C2'—Cr	−124.3 (2)	C4'—Cr—C5'—C6'	131.2 (2)
C11—Cr—C2'—C3'	−123.62 (17)	C11—Cr—C5'—C4'	−96.4 (4)
C12—Cr—C2'—C3'	−36.9 (2)	C12—Cr—C5'—C4'	60.89 (17)
C13—Cr—C2'—C3'	154.20 (18)	C13—Cr—C5'—C4'	152.48 (16)
C6'—Cr—C2'—C3'	101.74 (17)	C2'—Cr—C5'—C4'	−64.95 (15)
C1'—Cr—C2'—C3'	131.5 (2)	C6'—Cr—C5'—C4'	−131.2 (2)
C5'—Cr—C2'—C3'	65.04 (16)	C1'—Cr—C5'—C4'	−102.40 (16)
C4'—Cr—C2'—C3'	29.07 (15)	C3'—Cr—C5'—C4'	−28.27 (14)
C11—Cr—C2'—C1'	104.87 (16)	C10—C5—C6—C7	−1.7 (4)
C12—Cr—C2'—C1'	−168.40 (16)	C4'—C5'—C6'—C1'	−0.4 (4)
C13—Cr—C2'—C1'	22.7 (2)	Cr—C5'—C6'—C1'	−55.7 (2)
C6'—Cr—C2'—C1'	−29.77 (14)	C4'—C5'—C6'—Cr	55.4 (2)
C3'—Cr—C2'—C1'	−131.5 (2)	C2'—C1'—C6'—C5'	2.8 (4)
C5'—Cr—C2'—C1'	−66.47 (15)	C3—C1'—C6'—C5'	−179.4 (2)
C4'—Cr—C2'—C1'	−102.44 (16)	Cr—C1'—C6'—C5'	56.1 (2)
C1'—C2'—C3'—C4'	1.5 (4)	C2'—C1'—C6'—Cr	−53.3 (2)
Cr—C2'—C3'—C4'	−55.2 (2)	C3—C1'—C6'—Cr	124.6 (2)
C1'—C2'—C3'—Cr	56.7 (2)	C11—Cr—C6'—C5'	−167.85 (15)
C11—Cr—C3'—C2'	60.24 (18)	C12—Cr—C6'—C5'	19.0 (2)
C12—Cr—C3'—C2'	151.31 (17)	C13—Cr—C6'—C5'	105.92 (16)
C13—Cr—C3'—C2'	−100.7 (4)	C2'—Cr—C6'—C5'	−102.57 (16)
C6'—Cr—C3'—C2'	−66.83 (16)	C1'—Cr—C6'—C5'	−132.7 (2)
C1'—Cr—C3'—C2'	−29.73 (15)	C3'—Cr—C6'—C5'	−65.54 (15)
C5'—Cr—C3'—C2'	−103.53 (17)	C4'—Cr—C6'—C5'	−29.31 (14)
C4'—Cr—C3'—C2'	−131.7 (2)	C11—Cr—C6'—C1'	−35.1 (2)
C11—Cr—C3'—C4'	−168.06 (16)	C12—Cr—C6'—C1'	151.72 (18)
C12—Cr—C3'—C4'	−76.99 (17)	C13—Cr—C6'—C1'	−121.35 (16)
C13—Cr—C3'—C4'	31.0 (5)	C2'—Cr—C6'—C1'	30.16 (14)
C2'—Cr—C3'—C4'	131.7 (2)	C3'—Cr—C6'—C1'	67.20 (15)
C6'—Cr—C3'—C4'	64.87 (16)	C5'—Cr—C6'—C1'	132.7 (2)
C1'—Cr—C3'—C4'	101.97 (17)	C4'—Cr—C6'—C1'	103.42 (16)
C5'—Cr—C3'—C4'	28.17 (15)	C5—C6—C7—O7	−177.2 (3)
O5—C2—C3—C4	−2.0 (4)	C5—C6—C7—C8	3.3 (4)
O5—C2—C3—C1'	179.2 (2)	O7—C7—C8—C9	179.0 (3)
C6'—C1'—C3—C2	142.3 (3)	C6—C7—C8—C9	−1.6 (4)
C2'—C1'—C3—C2	−39.9 (4)	C7—C8—C9—O5	179.3 (2)
Cr—C1'—C3—C2	−127.5 (2)	C7—C8—C9—C10	−1.8 (4)
C6'—C1'—C3—C4	−36.6 (3)	O5—C9—C10—C5	−177.9 (2)
C2'—C1'—C3—C4	141.3 (2)	C8—C9—C10—C5	3.3 (4)
Cr—C1'—C3—C4	53.6 (3)	O5—C9—C10—C4	4.3 (4)
C2—C3—C4—O4	−172.4 (3)	C8—C9—C10—C4	−174.6 (2)
C1'—C3—C4—O4	6.4 (4)	C6—C5—C10—C9	−1.5 (4)
C2—C3—C4—C10	7.1 (4)	C6—C5—C10—C4	176.3 (3)
C1'—C3—C4—C10	−174.1 (2)	O4—C4—C10—C9	171.3 (3)
C2'—C3'—C4'—O4'	179.6 (2)	C3—C4—C10—C9	−8.3 (4)
Cr—C3'—C4'—O4'	125.8 (2)	O4—C4—C10—C5	−6.5 (4)
C2'—C3'—C4'—C5'	1.1 (4)	C3—C4—C10—C5	174.0 (2)
Cr—C3'—C4'—C5'	−52.7 (2)	C5'—C4'—O4'—C41'	15.9 (4)
C2'—C3'—C4'—Cr	53.8 (2)	C3'—C4'—O4'—C41'	−162.6 (2)
C11—Cr—C4'—O4'	−88.0 (3)	Cr—C4'—O4'—C41'	−77.7 (3)
C12—Cr—C4'—O4'	−3.0 (2)	C3—C2—O5—C9	−2.6 (4)
C13—Cr—C4'—O4'	83.4 (3)	C10—C9—O5—C2	1.4 (4)
C2'—Cr—C4'—O4'	−136.3 (3)	C8—C9—O5—C2	−179.7 (2)
C6'—Cr—C4'—O4'	149.5 (3)	C8—C7—O7—C71	−5.5 (4)
C1'—Cr—C4'—O4'	−173.8 (2)	C6—C7—O7—C71	175.0 (3)
C3'—Cr—C4'—O4'	−106.9 (3)		
